# Amygdala volume is not associated with MRI-based markers of early cardiovascular disease

**DOI:** 10.1186/s13244-025-02190-4

**Published:** 2026-01-26

**Authors:** Sarah Schlaeger, Roberto Lorbeer, Fabian Bamberg, Christopher L. Schlett, Susanne Rospleszcz, Ebba Beller, Franziska Galie, Margit Heier, Karl-Heinz Ladwig, Jens Ricke, Annette Peters, Birgit B. Ertl-Wagner, Sophia Stoecklein, Sergio Grosu

**Affiliations:** 1 Department of Radiology, LMU University Hospital, LMU Munich, Munich, Germany; 2 Department of Diagnostic and Interventional Radiology, Medical Center–University of Freiburg, Faculty of Medicine, University of Freiburg, Freiburg, Germany; 3 German Research Center for Environmental Health, Institute of Epidemiology, Helmholtz Center Munich, Neuherberg, Germany; 4 Institute of Diagnostic and Interventional Radiology, Pediatric Radiology and Neuroradiology, University Medical Center Rostock, Rostock, Germany; 5 KORA Study Centre, University Hospital of Augsburg, Augsburg, Germany; 6 Department of Psychosomatic Medicine and Psychotherapy, Klinikum rechts der Isar, Technical University Munich, Munich, Germany; 7 German Center for Cardiovascular Research, Partner Site Munich Heart Alliance, Munich, Germany; 8 Department of Epidemiology, Biometry and Epidemiology, Institute for Medical Information Processing, LMU Munich, Munich, Germany; 9 Department of Diagnostic Imaging, The Hospital for Sick Children, University of Toronto, Toronto, Canada; 10 German Center for Diabetes Research (DZD), Neuherberg, Germany; 11 Department of Medical Imaging, University of Toronto, Toronto, Canada

**Keywords:** Amygdala, Cardiovascular disease, Magnetic resonance imaging, Psychological stress

## Abstract

**Background:**

Recent PET studies suggest a link between amygdala activity and cardiovascular disease. Altered amygdala volumes are associated with increased stressor-evoked cardiovascular reactivity, which potentially increases the risk for cardiovascular disease. Therefore, we investigated the association between amygdala volume and MRI-based markers of cardiovascular disease in order to evaluate morphological alterations of the amygdala in persons with early, clinically inapparent signs of cardiovascular complications.

**Materials and methods:**

400 subjects underwent a comprehensive 3-T MRI to estimate amygdala volume and imaging-based markers of cardiovascular disease, specifically carotid plaque presence and grading, media wall thickening, left ventricular myocardial mass, myocardial late gadolinium enhancement, and left ventricular function. Amygdala volume was automatically segmented based on FLAIR images and corrected for total intracranial volume. Logistic and linear regression analyses of amygdala volume and cardiovascular parameters were conducted while controlling for age, gender and cardiovascular risk factors.

**Results:**

Among 339 included subjects (mean age: 56.3 ± 9.1, 57% males), the average absolute amygdala volume was 3.04 ± 0.24 mL, and the average amygdala ratio was 0.213 ± 0.017% of total intracranial volume. Carotid plaque was present in 22.6%, and myocardial late gadolinium enhancement in 3.2%. Mean media wall thickening was 0.76 ± 0.1 mm, mean left ventricular myocardial mass 71.6 ± 14.1 g/m^2^, and mean ejection fraction 69.1 ± 8.2%. Logistic and linear regression analyses showed no significant association of amygdala volume and any of the MRI-based cardiovascular parameters (*p* > 0.05, respectively).

**Conclusions:**

Amygdala volume was not associated with early MRI-based markers of cardiovascular disease, suggesting that the amygdala is not morphologically altered in the initial phase of cardiovascular disease.

**Critical relevance statement:**

This first large MRI study demonstrates that amygdala volume is not associated with subclinical cardiovascular disease, critically refining prior PET-based hypotheses and advancing clinical radiology by clarifying the preserved role of amygdala morphology in early cardiovascular pathology.

**Key Points:**

- PET studies link amygdala activity to cardiovascular disease, while the role of amygdala volume in early cardiovascular disease is unclear.
- In this large population-based study, 339 asymptomatic adults who underwent comprehensive 3-T MRI with cardiovascular assessment were analyzed.
- Amygdala volume showed no association with MRI markers of subclinical cardiovascular disease.
- This is the first large MRI study linking amygdala volume and early cardiovascular disease.

**Graphical Abstract:**

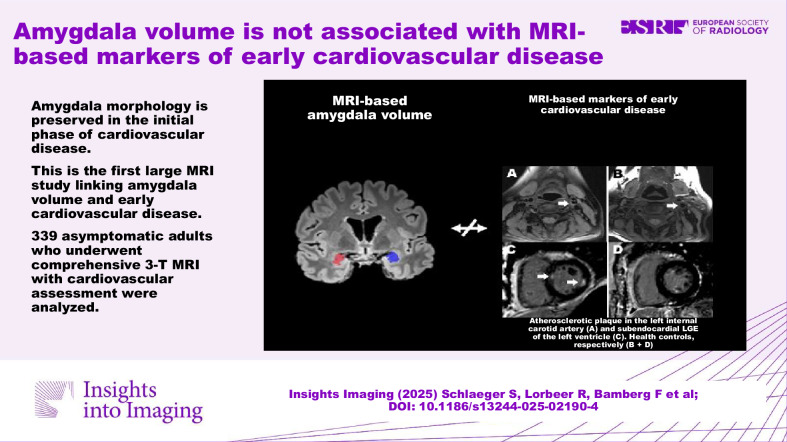

## Introduction

Cardiovascular disease (CVD) accounts for 31% of all deaths worldwide [1]. Amongst other risk factors like diabetes, blood cholesterol levels, hypertension, smoking and obesity, chronic stress is independently associated with an increased risk of CVD [2]. The attributable risk of chronic stress is equal to other major cardiovascular risk factors [3].

The brain’s salience network is thought to have an important role in transforming chronic stress into CVD. This intrinsically connected large-scale network, with the amygdala as a key component, is responsible for complex functions such as cognition and emotion [4]. Its activation triggers autonomic and hormonal responses in conditions marked by stress, such as post-traumatic stress disorder (PTSD), anxiety, and depression [5, 6]. Through the upregulation of hematopoietic tissue activity and increased atherosclerotic inflammation, amygdala function and connectivity seem to have an important linking role in the mechanisms translating stress into cardiovascular disease [7, 8].

Recent publications validated the relation between amygdala activity and CVD in ^18^F-FDG-PET/CT measurements and showed that amygdala activity was associated with a history of coronary artery disease [9], higher rates of cardiovascular events [10], with coronary inflammation and high-risk plaques [11] or independently and robustly predicted cardiovascular disease events [12]. In acute myocardial infarction, a linking interconnection between amygdala activity assessed with ^18^F-FDG-PET/CT, arterial inflammation and macrophage haematopoiesis was shown [13]. However, amygdala activity measurements using PET/CT require radiotracers, involve ionizing radiation, and remain limited in availability.

Alternatively, amygdala activity can be assessed using functional magnetic resonance imaging (fMRI). Individuals with cardiovascular risk markers, such as exaggerated stressor-evoked blood pressure responses and increased carotid intima-media thickness, exhibit elevated amygdala activation/reactivity on fMRI alongside stronger positive functional connectivity between amygdala and pons or the pregenual anterior cingulate cortex [8, 14]. Recently, Rasero et al demonstrated a link between stressor-evoked brain activity and preclinical atherosclerosis with the amygdala as part of the implicated brain areas in this mediation [15].

Next to alterations of amygdala activity, animal models have shown that stress leads to larger amygdala volumes by increased dendritic arborization [16, 17]. Moreover, in healthy subjects, increased stressor-evoked cardiovascular reactivity, which potentially increases the risk for CVD, was linked to reduced amygdala volumes. Specifically, Gianaros et al demonstrated that greater mean arterial pressure reactivity was associated with lower amygdala volumes, whereas in a second study, heart rate and cardiac output reactivity, but not mean arterial blood pressure reactivity, were connected to reduced amygdala volumes [14, 18]. In patients with HIV, amygdala volumes were reduced in individuals with a higher cardiovascular risk score [19].

Structural MRI is well-suited for assessing amygdala morphology because it is widely available, easily integrated into routine imaging, and applicable both for screening and opportunistic evaluation. Automated amygdala volumetry based on MRI is well established and enables reliable large-scale quantification [20].

Additionally, MRI provides non-invasive, high-resolution cardiac and vascular phenotyping, enabling the (simultaneous) assessment of markers of cardiovascular disease such as carotid plaque burden, media wall thickening, left ventricular (LV) myocardial mass, myocardial late gadolinium enhancement, and LV function.

While stress has been suggested as a possible link between alterations in amygdala activity/volume and CVD, the role of amygdala volume in early CVD is unclear.

Therefore, the aim of this study was to determine whether MRI-derived amygdala volume is associated with MRI-based markers of subclinical cardiovascular disease—including carotid plaque burden, carotid wall thickening, LV myocardial mass, myocardial late gadolinium enhancement, and LV function—in a population free of overt cardiovascular disease, thereby assessing whether amygdala morphology reflects early, preclinical cardiovascular alterations.

## Materials and methods

### Study design

The study comprises a prospective cohort from the Cooperative Health Research in the Region of Augsburg (KORA), which is a regional research platform for population-based studies. The study design, data collection and sampling method have been described in detail beforehand [21].

The study was performed in accordance with the Declaration of Helsinki, including written informed consent from all participants. All study methods were approved by the ethics committee of the Bavarian Chamber of Physicians, Munich (S4: EC No. 99186 and for genetic epidemiological questions 05004, F4 and FF4: EC No. 06068). The MRI examination protocol was approved by the ethics committee of the LMU University Hospital, Munich.

### Subject selection

We used data from the population-based KORA FF4 study (2013–2014, 2279 subjects). The FF4 study is the second follow-up of the baseline study KORA S4 (1999–2001, 4261 subjects), which is a large sample from the general population in the region of Augsburg, Germany [21]. The MRI study nested in FF4 included 400 participants, facilitating a case-control design of 54 subjects with established diabetes, 103 subjects with prediabetes, and the rest with normal glucose metabolism. All 400 subjects underwent a dedicated whole-body MRI protocol [22]. Exclusion criteria were: age > 74 years, validated/self-reported stroke, myocardial infarction, peripheral artery disease, type 1 diabetes mellitus, poor overall health condition, missing oral glucose tolerance test result or contraindications for MRI examinations.

### MRI

Image acquisition was performed on a single 3-Tesla MRI system (Magnetom Skyra; Siemens AG, Healthcare Sector) using an 18-channel body coil in combination with the table-mounted spine matrix coil and a 20-channel head and neck coil. Patients were scanned in a supine position on a flat table-top insert. The whole-body protocol comprised sequences to cover the brain as well as the cardiovascular system, i.e. 3D T2-weighted fluid-attenuated inversion recovery (T2w 3D-FLAIR) for brain imaging {slice thickness (ST): 0.9 mm, field of view (FOV): 245 × 245 mm, repetition time (TR): 5000 ms, echo time (TE): 389 ms, inversion time (TI): 1800 ms}; 4-chamber view steady state free precession (SSFP) and short-axis stack SSFP for cardiac imaging {ST: 8 mm, FOV: 297 × 360 mm, TR: 29.97 ms, TE: 1.46 ms}; fast low-angle shot (FLASH) for LGE {ST: 8 mm, FOV: 300 × 360 mm, TR: 700–1000 ms, TE: 1.55 ms, TI: 280–345 ms}; axial blackblood T1-weighted fat saturated for carotid plaque {ST: 3 mm, FOV: 165 × 220 mm, TR: 800 ms, TE: 13 ms}. Image analyses were performed blinded to all clinical data and other measurements by independent readers using dedicated off-line workstations.

### Amygdala volume

**Fig. 1:**
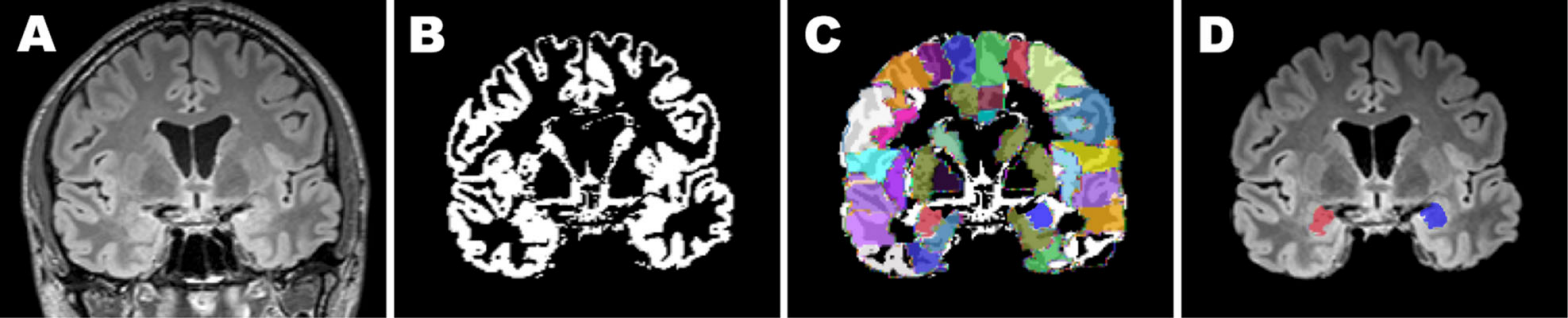
Example of automated warp-based segmentation of amygdala volumes. T2-weighted 3D-FLAIR datasets of the brain (**A**) were used for automated brain extraction (**B**). The obtained transformation matrix was inverted and warped onto the Automatic Anatomical Labeling atlas (**C**). The total volume of white matter, gray matter and cerebrospinal fluid, as well as the amygdala volume, was calculated. (**D**) Right amygdala marked in red, left amygdala marked in blue

Amygdala volumes were assessed on T2w 3D-FLAIR images using automated brain segmentation as described in detail previously (Fig. 1) [20]. Image pre-processing was performed using freely available software (FSL 5.0.9; FMRIB, University of Oxford, and AFNI; National Institute of Mental Health). After brain extraction, FLAIR images were reoriented and segmented into white matter, gray matter and cerebrospinal fluid using an automated segmentation tool (FAST; FMRIB, University of Oxford) [23]. The obtained transformation matrix was inverted and warped onto the Automatic Anatomical Labeling (AAL) atlas in MNI standard space, applying linear and non-linear registration as implemented in FLIRT and FNIRT [24]. Lastly, the total volume of white matter, gray matter and cerebrospinal fluid, as well as the amygdala volume, were calculated. The MRI images of the brain and the automatic segmentations were independently reviewed for quality by two board-certified radiologists with 7 and 2 years of experience. To ensure a homogenous dataset, any case in which at least one reader judged the MRI image quality or the amygdala segmentation quality as insufficient was excluded from the analysis (*n* = 49 excluded out of *n* = 400 subjects). Total intracranial volume (ICV) was calculated by summing up white matter, gray matter and cerebrospinal fluid volumes and used for the normalization of amygdala volumes [25]. A ratio-based normalization approach for amygdala volume (aVol) was applied (amygdala ratio (%) = [aVol (mL) / ICV (mL)] × 100) in order to adjust for differences in head size. Right amygdala volumes, left amygdala volumes and total amygdala volumes were calculated.

### Carotid media wall thickening and plaque

**Fig. 2:**
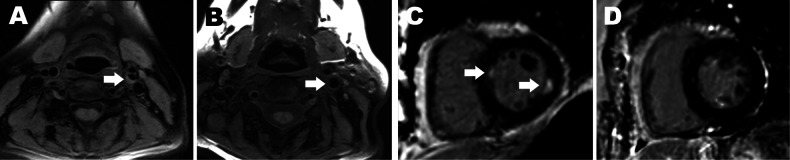
Example of MRI-based assessment of CVD-markers. Atherosclerotic plaque in the left internal carotid artery (arrow, **A**) compared to a healthy control subject without visualizable atherosclerotic plaque (arrow, **B**) and subendocardial LGE of the left ventricle (arrows, **C**) compared to a healthy control subject with no evidence of ischemic enhancement (**D**)

Media wall thickening, presence of carotid plaque, plaque burden and composition of carotid plaques were assessed manually on fat-suppressed, T1-weighted, blackblood images by one radiologist with 5 years of experience as described in detail previously (Fig. 2) [22]. Measurements were made of both sides of the distal common carotid artery, of the carotic bulb, and of the proximal internal carotid artery, according to previously published criteria using histology as the gold standard [26]. Boundaries of the vessel lumen and the vessel wall were determined semi-automatically using commercially available software (CASCADE; University of Washington). Manual corrections were performed if necessary. The presence of calcification and hemorrhage, wall thickness and wall eccentricity were class-divided according to the modified American Heart Association (AHA) classification of atherosclerotic plaque for MRI in type I/II, type III, type IV/V, and type VI/VII plaques. Each side was classified separately. Repeated measurements of media wall thickness demonstrated an intra-reader variability of relative difference < 5%.

### Late gadolinium enhancement

The presence and characteristics of late gadolinium enhancement (LGE) were determined by two radiologists with 2 and 5 years of experience, on FLASH sequences in short-axis stacks and a four-chamber view using commercially available software (cvi42; Circle Cardiovascular Imaging) as described in detail beforehand (Fig. 2) [22]. Distribution patterns were class-divided according to the AHA 17-segment model in subendocardial, midmyocardial, and epicardial [27]. Consensus reading was performed in case of discrepancy.

### Left ventricular mass and function

LV function was evaluated semi-automatically, using commercially available software (cvi42; Circle Cardiovascular Imaging) by two radiologists with 2 and 5 years of experience, on SSFP sequences as described in detail previously [22]. Automatic LV-endocardium contour detection was performed for the assessment of LV myocardial mass. Manual corrections were performed if necessary, according to current guidelines [28], by the same two radiologists as described above. Filling and ejection rates were calculated using associated gradients and time lags by using dedicated in-house software displaying the LV volume versus the time curve and its derivative. Peak gradients due to atrial contraction during early, passive LV filling and late LV filling are estimated, as described previously [29]. Reproducibility studies showed low inter-reader variability with relative differences of < 9% for left ventricular mass, < 5% for LV volumes, as well as LV ejection fraction.

### Statistical analyses

Continuous variables are described as arithmetic means with standard deviation or medians with 1st and 3rd quartile. Categorical variables are presented as counts and percentages. Characteristic differences were evaluated by *t*-test, chi^2^-test or one-way analysis of variance, and *p*-values were calculated.

Linear and logistic regression models were used to evaluate the associations between amygdala volume and subclinical MRI-based markers of cardiovascular disease. Adjusted β-coefficients and odds ratios (OR) with 95% confidence intervals (CI) are provided. Associations of amygdala volume and subclinical MRI-based markers of cardiovascular disease were adjusted for age, gender, smoking, diabetes mellitus, LDL cholesterol level, body mass index (BMI) (Model A) and additionally for systolic blood pressure (Model B) to account for potential confounding bias. A *p*-value of less than 0.05 was considered statistically significant with regard to all analyses. Statistical analyses were performed using the Stata 14.1 software package (Stata Corporation).

## Results

### Study population

Sample sizes differed between amygdala volume (*n* = 351) and cardiovascular exposure groups of carotid media wall thickening (*n* = 261), carotid plaque (*n* = 230), LV myocardial mass (*n* = 339), LGE (*n* = 339) and cardiac function (*n* = 339) due to either insufficient image quality or missing imaging data. All of the above MRI measurements were available in 216 subjects.

The maximal final study population consisted of 339 subjects (57% male). Mean age was 56.3 (± 9.1) years (57% male). Further details are presented in Table 1.

**Table 1:** Characteristics of the study population stratified by gender

	Total *n* = 339	Women *n* = 146	Men *n* = 193	*p*-value*
Age (years)	56.3 (± 9.1)	56.2 (± 9.1)	56.3 (± 9.1)	0.886
Weight (kg)	82.3 (± 16.3)	73.1 (± 15.1)	89.1 (± 13.7)	< 0.001
Height (cm)	171.6 (± 9.7)	163.5 (± 6.6)	177.8 (± 6.7)	< 0.001
BMI (kg/m^2^)	27.9 (± 4.8)	27.4 (± 5.6)	28.2 (± 4.2)	0.131
Total cholesterol (mg/dL)	217.9 (± 37.1)	220.6 (± 35.8)	215.9 (± 38.1)	0.247
HDL (mg/dL)	62.1 (± 18)	71.2 (± 18.2)	55.2 (± 14.6)	< 0.001
LDL (mg/dL)	140.1 (± 33)	137.8 (± 32.8)	141.9 (± 33.2)	0.264
Triglycerides (mg/dL)	129.2 (± 82.6)	100.0 (± 42.3)	151.3 (± 97.6)	< 0.001
Physical activity in categories				0.062
1 = regularly more than 2 h/week	99 (29.2%)	39 (26.7%)	60 (31.1%)	
2 = regularly ca. 1 h/week	111 (32.7%)	56 (38.4%)	55 (28.5%)	
3 = irregularly ca. 1 h/week	50 (14.8%)	25 (17.1%)	25 (13%)	
4 = little or no physical activity	79 (23.3%)	26 (17.8%)	53 (27.5%)	
Smoking status				0.041
Never smoker	123 (36.3%)	62 (42.5%)	61 (31.6%)	
Former smoker	144 (42.5%)	51 (34.9%)	93 (48.2%)	
Current smoker	72 (21.2%)	33 (22.6%)	39 (20.2%)	
Pack years	13.2 (± 18.4)	8.3 (± 12.4)	17 (± 21.2)	< 0.001
Diabetes mellitus	42 (12.4%)	12 (8.2%)	30 (15.5%)	0.043
Systolic BP (mmHg)	120.7 (± 17.2)	113.1 (± 14.8)	126.5 (± 16.6)	< 0.001
Diastolic BP (mmHg)	75.4 (± 10.3)	71.9 (± 8.6)	78.0 (± 10.7)	< 0.001
Hypertension	113 (33.3%)	39 (26.7%)	74 (38.3%)	0.024
Antihypertensive medication	81 (23.9%)	37 (25.3%)	44 (22.8%)	0.586
Lipid-lowering medication	32 (9.4%)	12 (8.2%)	20 (10.4%)	0.504
Amygdala volume ratio (amygdala volume in relation to ICV)				
Total (%)	0.213 (± 0.017)	0.215 (± 0.016)	0.212 (± 0.018)	0.265
Right (%)	0.105 (± 0.008)	0.105 (± 0.008)	0.105 (± 0.008)	0.712
Left (%)	0.109 (± 0.01)	0.110 (± 0.009)	0.108 (± 0.01)	0.111
Carotid plaque				
Presence of plaque	52 (22.6%)	19 (21.8%)	33 (23.1%)	0.828
Plaque type				0.932
AHA/MRI type I	183 (83.2%)	69 (81.2%)	114 (84.4%)	
AHA/MRI type III	28 (12.7%)	12 (14.1%)	16 (11.9%)	
AHA/MRI type V	7 (3.2%)	3 (3.5%)	4 (3%)	
AHA/MRI type VI or VII	2 (0.9%)	1 (1.2%)	1 (0.7%)	
Carotid media wall thickening				
Wall thickness, LCA (mm)	0.75 (± 0.11)	0.73 (± 0.11)	0.76 (± 0.11)	0.083
Wall thickness, RCA (mm)	0.76 (± 0.1)	0.74 (± 0.1)	0.77 (± 0.1)	0.037
LV myocardial mass (g/m^2^)	71.6 (± 14.1)	63.7 (± 11.4)	77.5 (± 13.1)	< 0.001
Late gadolinium enhancement	11 (3.2%)	2 (1.4%)	9 (4.7%)	0.090
Cardiac function				
End-diastolic volume (mL/m^2^)	66.7 (± 15.1)	65.8 (± 13.3)	67.3 (± 16.4)	0.366
End-systolic volume (mL/m^2^)	21.0 (± 8.7)	19.4 (± 8)	22.1 (± 9)	0.004
Stroke volume (mL/m^2^)	45.7 (± 10)	46.4 (± 9.3)	45.2 (± 10.4)	0.266
Ejection fraction (%)	69.1 (± 8.2)	70.9 (± 7.9)	67.8 (± 8.1)	< 0.001
Peak ejection rate (mL/s)	355.8 (± 136)	330.1 (± 108.3)	375.1 (± 151)	0.003

Values in mean ± standard deviation or number and percentage

* *p*-values are from *t*-test or chi^2^-test

### Amygdala volume

Mean absolute amygdala volume was 3.04 (± 0.24) mL (female: 3.05 (± 0.22) mL, male: 3.03 (± 0.26) mL). Mean amygdala ratio to ICV was 0.213 (± 0.017) % (female: 0.215 (± 0.016) %, male: 0.212 (± 0.018) %). There were no significant differences in amygdala measurements between men and women. Further details are presented in Table 1.

### Cardiovascular findings

Carotid plaque was present in 52 (22.6%) subjects, and myocardial LGE in 11 (3.2%) subjects. Mean carotid media wall thickness was 0.76 (± 0.1) mm, mean myocardial mass 71.6 (± 14.1) g/m^2^. Mean ejection fraction was 69.1 (± 8.2) %, mean end-diastolic volume 66.7 (± 15.1) mL/m^2^, mean end-systolic volume 21.0 (± 8.7) mL/m^2^, mean stroke volume 45.7 (± 10) mL/m^2^ and mean peak ejection rate was 355.8 (± 136) mL/m^2^. Further details are presented in Table 1.

### Association of amygdala volume and cardiovascular findings

After adjustment for age, gender, smoking, diabetes mellitus, LDL cholesterol level, BMI (Model A) and additionally for systolic blood pressure (Model B), logistic and linear regression analyses showed no significant association of amygdala volume and any of the cardiovascular parameters (*p* > 0.05, respectively). Further details are presented in Table 2.

**Table 2:** Association of amygdala volume and MRI-based markers for cardiovascular disease (CVD)

Association of amygdala volume with carotid plaque (*n* = 230)			
	Amygdala volume (%) total	Amygdala volume (%) right	Amygdala volume (%) left
	OR (95% CI)	OR (95% CI)	OR (95% CI)
Presence of plaque	0.90 (0.61; 1.32)	1.02 (0.71; 1.48)	0.82 (0.56; 1.20)
AHA/MRI plaque type	0.82 (0.54; 1.26)	0.99 (0.65; 1.49)	0.73 (0.48; 1.11)
Association of amygdala volume with carotid media wall thickening (*n* = 261)			
	Amygdala volume (%) total	Amygdala volume (%) right	Amygdala volume (%) left
	β (95% CI)	β (95% CI)	β (95% CI)
Wall thickness (mm) LCA	−0.004 (−0.019; 0.011)	0.004 (−0.011; 0.018)	−0.01 (−0.025; 0.005)
Wall thickness (mm) RCA	0.004 (−0.01; 0.018)	0.011 (−0.002; 0.025)	−0.003 (−0.018; 0.011)
Association of amygdala volume with LV myocardial mass (*n* = 339)			
	Amygdala volume (%) total	Amygdala volume (%) right	Amygdala volume (%) left
	β (95% CI)	β (95% CI)	β (95% CI)
LV myocardial mass (g/m^2^)	0.466 (−0.83; 1.761)	0.835 (−0.465; 2.134)	0.106 (−1.188; 1.399)
Association of amygdala volume with LGE (*n* = 339)			
	Amygdala volume (%) total	Amygdala volume (%) right	Amygdala volume (%) left
	OR (95% CI)	OR (95% CI)	OR (95% CI)
LGE	0.623 (0.335; 1.157)	0.616 (0.337; 1.125)	0.663 (0.353; 1.245)
Association of amygdala volume with cardiac function (*n* = 339)			
	Amygdala volume (%) total	Amygdala volume (%) right	Amygdala volume (%) left
	β (95% CI)	β (95% CI)	β (95% CI)
End-diastolic volume (mL/m^2^)	−0.338 (−1.891; 1.215)	−0.568 (−2.128; 0.991)	−0.108 (−1.657; 1.442)
End-systolic volume (mL/m^2^)	−0.112 (−1.029; 0.804)	−0.125 (−1.046; 0.796)	−0.088 (−1.003; 0.826)
Stroke volume (mL/m^2^)	−0.24 (−1.261; 0.78)	−0.447 (−1.472; 0.578)	−0.042 (−1.06; 0.977)
Ejection fraction (%)	0.167 (−0.702; 1.036)	0.113 (−0.76; 0.987)	0.191 (−0.676; 1.058)
Peak ejection rate (mL/s)	2.954 (−11.051; 16.959)	6.195 (−7.871; 20.261)	−0.065 (−14.044; 13.914)

Results of logistic and linear regression models adjusted for age, gender, smoking, diabetes mellitus, LDL-C, BMI and systolic blood pressure. β-coefficients are from linear regression models. Odds ratios (OR) are from logistic regression models

## Discussion

In the present study, we investigated the association between amygdala volume and MRI-based markers of CVD in a population without clinically apparent CVD in order to evaluate morphological changes of the amygdala before clinical symptoms occur. Absolute and ICV-corrected amygdala volumes were in keeping with volumes reported in current literature [30, 31]. Our results showed that amygdala volume was not associated with MRI-based markers of CVD such as estimates of carotid plaque, media wall thickening, LV myocardial mass, myocardial late gadolinium enhancement or LV function.

Amygdala activity, measured by ^18^F-FDG-PET/CT, has been reported to independently and robustly predict cardiovascular disease events. It has furthermore been demonstrated that increased amygdala activity is associated with increased hemopoietic activity and increased arterial inflammation, which could be detected as a correlated signal increase in ^18^F-FDG-PET/CT measurements. That is to say, resting metabolic activity within the amygdala measured in ^18^F-FDG-PET/CT robustly predicts the development of CVD, independent of proven cardiovascular risk factors. Consequently, the amygdala could be a key structure in the mechanisms linking stress to CVD [12].

Increased amygdala metabolism might lead to amygdala enlargement [32, 33]. Yet, greater amygdala activity and reduced amygdala volumes were associated with increased stressor-evoked cardiovascular reactivity, which potentially increases the risk for CVD [14, 18].

Stress has been suggested as a possible link between alterations in amygdala activity and volume and CVD. However, human data about the relation between amygdala volume and stress remain inconclusive. Early childhood stress exposure has been associated with increased amygdala volume in some studies [34–37], while other studies did not report any significant effect on amygdala volume [38, 39]. Stress caused by trauma resulting in PTSD or early-life stress, such as experiencing low socioeconomic status, might even lead to a decrease in amygdala volume [13, 40–42]. Variations in amygdala volume of individuals exposed to stress could be caused by several factors. The timing and type of stress exposure seem to play an important role. Prior studies found that amygdala volume may be especially sensitive to structural changes during early childhood when it is rapidly growing, as well as during preadolescence when its growth peaks [43, 44]. Yet, the effects of lifestyle-associated stress factors that typically occur later in life, such as a high workload, sleep deprivation or stress caused by time constraints on amygdala volume have not yet been conclusively assessed. Consequently, amygdala volume as a link between stress and CVD appears to be influenced by numerous potentially interrelated factors with partially opposite effects, which may partly explain the outcome of this study.

The following limitations to our study need to be taken into account when interpreting the data. First, although associations of amygdala volume and subclinical MRI-based markers of CVD were adjusted for age, gender, smoking, diabetes mellitus, LDL cholesterol level, BMI and systolic blood pressure, additional potential confounders of amygdala volume such as early-life stress, neuropsychiatric conditions (PTSD, bipolar disorder, acute state of depression, autism) cocaine addiction, or lithium medication were not taken into account [45–48]. These unmeasured factors are known to influence brain morphology and therefore represent a limitation to interpreting the volumetric analysis. Second, the MRI data were originally acquired as a nested case-control study to investigate primarily the differences in subclinical CVD between subjects with diabetes, prediabetes and non-diabetic controls. Consequently, the MRI sub-study was enriched with prediabetic and diabetic subjects. Although additional analyses using weights accounting for dissimilarities between the aforementioned MRI sub-study and the whole underlying cohort did not reveal any differences [49], our findings are most generalizable to a population at high cardiometabolic risk, which may differ from a perfectly healthy population. Third, the prevalence of myocardial LGE as an MRI-based marker of CVD was low, with just 3.2% (11 subjects). Although this low prevalence is expected because our study cohort consisted of individuals free of overt cardiovascular disease, this significantly reduces the statistical power to detect an association with this specific marker, and therefore, this result must be interpreted with caution. Fourth, amygdala volume was measured based on T2-weighted 3D-FLAIR images using automated brain segmentation. Although most established segmentation pipelines rely on T1-weighted acquisitions, protocol constraints in the whole-body setting did not allow for additional 3D T1-weighted brain imaging. We therefore performed a validation study of this FLAIR-based approach, which showed a good correlation of amygdala volumes based on T1-weighted images and amygdala volumes based on FLAIR images (Pearson correlation: r = 0.68–0.72) [20]. Nevertheless, the use of FLAIR-based automated segmentation may introduce measurement bias relative to T2-weighted ground truth segmentation and has to be considered a methodological limitation.

## Conclusion

In this population without clinically apparent CVD, amygdala volume was not associated with MRI-based markers of CVD such as carotid plaque presence and grading, media wall thickening, LV myocardial mass, myocardial late gadolinium enhancement or LV function. Our results suggest that the amygdala is not morphologically altered in the initial phase of CVD.

## Data Availability

The data underlying this article will be shared on reasonable request to the corresponding author.

## Abbreviations

AHA: American Heart Association
aVol: Amygdala volume
BMI: Body mass index
CI: Confidence intervals
CT: Computed tomography
CVD: Cardiovascular disease
FDG: Fluordesoxyglucose
FLAIR: Fluid-attenuated inversion recovery
FLASH: Fast low-angle shot
fMRI: Functional magnetic resonance imaging
FOV: Field of view
ICV: Intracranial volume
KORA: Cooperative health research in the region of Augsburg
LCA: Left carotid artery
LGE: Late gadolinium enhancement
LV: Left ventricular
MRI: Magnetic resonance imaging
OR: Odds ratios
PET: Positron-emission tomography
PTSD: Post-traumatic stress disorder
RCA: Right carotid artery
SSFP: Steady state free precession
ST: Slice thickness
TE: Echo time
TI: Inversion time
TR: Repetition time
